# Accelerating Innovation: Complexity, Regulation, and Temporality

**DOI:** 10.3389/fsoc.2019.00013

**Published:** 2019-03-12

**Authors:** Andrew Webster

**Affiliations:** Science and Technology Studies Unit, University of York, York, United Kingdom

**Keywords:** acceleration, innovation, regulation, risk, regenerative medicine

## Abstract

This Perspective paper explores recent moves seen in many countries toward accelerating the speed at which biomedical innovation can be delivered to the clinic. It discusses the drivers behind this and the rationale for it, illustrating this briefly in the field of regenerative medicine. It argues that the process reconfigures present/future relations, especially in terms of the relationship between different forms of evidence and risk calculations. The regulatory/innovation relationship is, as a result, being rewritten. Paradoxically, the moves toward acceleration are less to do with the arrival of a more streamlined evaluation system that selects for scientifically robust technologies ready for “the market.” In contrast, it reflects the growing complexity of innovation itself: whereas Latour (1987) had argued that “science-in the-making” was backstage in contrast to “ready-made science,” the former is now very visible. This in turn has generated two other parallel processes—“regulation-in-the-making” and “risk-in-the-making.” Such shifts can be seen in the field of regenerative medicine. The paper asks how best to engage with the move toward acceleration and thereby the future oversight of innovation.

## Introduction

The Research Topic for this special issue of *Frontiers in Sociology* asks us to consider how technology and the expertise on which it is based frame questions of risk and decision-making at different levels of the health system, both “upstream” and “downstream” in the innovation process. This Perspective piece argues that because of an increasingly complex socio-technical innovation system producing equally complex forms of scientific production and implementation, the upstream/downstream divide becomes less relevant, indeed misleading. I also suggest that this is directly associated with the widespread move toward accelerating innovation through more permissive regulatory regimes, especially within biomedicine. On the face of it, this is something of a paradox, since one might expect that the more complex and uncertain the science the slower one should go in moving toward its application. But this paradox can be resolved by rethinking how science is “made” today and how this in turn is producing a new way of making regulation itself.

## Complexity in Science

Science today can be characterized as being increasingly complex. This is the case in regard to the organization and production of science. Large-scale collaboration across institutions, technology platforms and disciplines (Vermeulen, 2016), the construction of digital platforms for data generation and interrogation, and the creation of international registers and repositories requiring the development of new standards characterize science production today. Allied to this is the development of new forms of regulatory oversight and economic evaluation. These developments are especially marked in the biomedical sciences (Cambrosio et al., 2018) and they not only change science practice and its coordination but create new socio-technical relations. The digitalization of biomedical science for example via “big data” does not simply change existing practices into “digital” ones but produces new types of social relations and organizational forms. As Barry (2001) has argued, the novelty of a technology lies as much in what new social relations it creates as it does any technical innovation *per se*. It also makes possible new questions, for example understanding the relation between the genomic, epigenomic and cellular levels, now possible to explore through data-intensive technological platforms (within both “wet” and computational biology). Indeed complexity *itself* becomes a topic of inquiry in science through the availability of fast and powerful computation. The bio and the info- together create new possibilities for life and its “regenesis” (Tamminen and Deibel, 2018).

A related, more broadly based, development has been the emergence of the “bioeconomy” as both a material project—generative of bio-commercial value and products—and a discursive one—generative of hype and hopes for the economy and society (Martin, 2015). As Marris (2018) observes, “…the bioeconomy is not simply “powered by” biological research and innovation: the bioeconomy *transforms* the organization and conduct of research and innovation that it purportedly depends on” (p. 58 emphasis in the original). High-level government bodies and funding agencies are promoting bioeconomic activity, as seen in the UK's life sciences industrial strategy (Office for Life Sciences, 2017). This emphasizes the importance of bringing public and private sector actors together, not just universities and industry (long a feature of research activity) but the NHS too, now positioned as an innovative partner in all this. The production of biomedicine becomes then increasingly hybridized across organizations in order to encourage faster and more effective innovation.

Complexity is found, then, in the questions that can be asked by science through its ever-increasing technical sophistication and epistemic density (which, in historical terms has perhaps always been one of the key features of science Shinn, 1999). Added to this, is the diversity of those who are involved in asking these questions, and the industrial scale of the international platforms that make such questions possible in the first place. Accountability, measures of success, and payment structures become simultaneously more complex, and to a degree difficult to manage and police, as a recent OECD report on genomics shows (Garden and Winickoff, 2018). Does this suggest that the way science is being produced and practiced is changing?

Thirty years ago the French sociologist of science Bruno Latour (1987) drew a useful distinction between “science-in-the making” and “ready-made-science.” Without oversimplifying too much, the former refers to the messy and serendipitous world of lab-science, yet to be prepared and polished for ready-made science, the world of the authoritative textbook, the classical experiment, the go-to model and method. The uncertainty of lab practices contrasts with the rhetorical claims of codified, factual science and the practices (institutional, network-based, and bureaucratic) that need to be undertaken to produce it. The two sides of science—the two sorts of practices of “science-in-action”—comprise its “Janus-faced” nature. There have been important criticisms of Latour's original argument (see e.g., Amsterdamska, 1990), in regard to both its characterization of science and its implications for what we can actually say about science. Even so, what we can retrieve perhaps is his focus on practice, the “making” of science, and indeed many scholars in STS have devoted considerable time and effort much more recently to explore science as practice (e.g., Pickering, 2010). Equally valuable was the contribution by (Nowotny et al., 2001) to describe the wider “Mode 2” form of scientific production, stressing in particular the epistemic shift in science toward a post-positivist reflexivity and recognition of uncertainty.

Taking such arguments a little further, we can suggest that the forms of complexity seen in science today make that science more explicitly provisional such that “science-in-the-making” occupies front of stage. It is not embarrassed to do so either, since the provisionality of production itself requires a new way of making science. This is exemplified by the significant shift toward open data sharing that not only build but also enable the interrogation of complex datasets: we see this in the sharing of genomic sequence data (across both academic and industrial players) to increase its use, reproducibility and economic value. Scientific “products”—such as an iPS cells—become and in some ways *only exist as distributed* objects, across datasets, standards, and organizational domains (Morrison, 2017). As a consequence, risk and decision-making are themselves distributed across a diverse range of actors: MacKenzie's (1998) famous “certainty trough” which showed how outside of those supportive of and those critical of a technology, most (users) in the “trough” tend to presume it to be stable and relatively risk-free or certain in its functionality and effects. Today, that trough is becoming less u-shaped and flatter as more diverse actors, comprising many publics, public and private sector producers and regulatory agencies, engage with science. This dissolves the traditional boundaries within which it is produced, opening new spaces for innovation and so a redistribution of risk across multiple actors.

From a science policy perspective, this complexity and uncertainty might be handled in various ways. Uncertainty may be explicitly acknowledged and publics informed that this is in fact the very nature of science, as President of the UK's Royal Society, Bob May, argued (May, 1997). Complexity may be actively embraced in the very framing of science as its development and potential impact are considered, as we have seen (Stilgoe et al., 2013) with the turn toward “responsible research and innovation” (RRI). As Felt (2017) has argued, however, RRI in principle can only be put into practice where it is possible for science producers to be “response-able,” that is to find that the organizational structures within which they work enable RRI to be practiced. In tandem with such moves, we have seen the creation of safe havens for new, contested science.

## Complexity, Risk, and Regulation

If this general characterization of an especially complex and uncertain science is reasonable, a number of questions arise: how do more complex, emergent areas of inquiry arise and where does their locus of expertise reside? How are standardized measures and their associated evidence-base constructed and by whom? What count as anomalous results where metrics are still being fashioned? How do the artifacts of science reflect complexity and how far can this be black-boxed, and indeed how transparent and thereby accountable can this process actually be? And, what role does regulatory science play in this regard? And with what political effects (Barry, 2001)?

It is this last point that I want to focus on here. Novel technologies pose a challenge to extant regulatory (and legal) knowledge and practice: two responses are seen, either an attempt to accommodate novelty via stretching existing regulations or developing new approaches that break with such conventions (Faulkner and Poort, 2017). Both are seen especially, though not exclusively, in the field of biomedicine (Faulkner, 2017). As the report *Making Perfect Life*, prepared for the European Parliament, observed:

“Almost all bio-engineering fields … challenge current regulatory policies. New types of interventions in the human body and brain force policy makers to anticipate new issues in the field of safety, privacy, bodily and mental integrity, and informed consent. New bio-, cogno-, and socio-inspired artifacts also lead to new safety, privacy and liability issues” (STOA, 2014).

As (Faulkner, 2017) has argued this has encouraged a “wait-and-see” risk sharing approach in the regulatory oversight of novel technologies (as process or product). In a similar vein, Zeitlin (2015) notes the arrival of “experimentalist governance,” described as “a recursive process of provisional goal-setting and revision based on learning from the comparison of alternative approaches to advancing them in different contexts” (p. 169). In biomedicine and elsewhere this reflects a wider development of the move toward risk-based regulation—a shift from prescriptive to performance-based approaches. Consequently, there has been a move to explore risk-sharing schemes that allow the quantification and management of immature evidence (for regulators) and allow the progressive collection of evidence for products. This recognizes the difficulties of managing complex, uncertain science as well as the desire, despite this, to make this highly provisional understanding the basis on which new products, techniques, therapies can be made available to the market early on. In Japan, for example, we have seen the introduction (in 2014) of its SAKIGAKE fast track approval system for prospective regenerative medicine therapies, which can be marketed and used as long as they have been shown to be safe in early phase trials. Measures of their actual efficacy are provided to regulators retrospectively in light of more data derived from those on the trial. Not surprisingly, this has attracted considerable interest from companies outside of Japan since conditional marketing authorization for up to 7 years can be given. This means they can be reimbursed while testing continues. The Japanese case reflects a wider trend toward accelerating the innovation/regulation nexus and a new distribution and definition of risk, as well as the mobility of global “biocapital” (Rajan, 2008) to exploit and cut across local regulatory regimes. The SAKIGAKE policy also carries bi-temporal effects in its conjoining future therapeutic promise with present-day practice. I shall explore these broad issues through a brief discussion of regenerative medicine.

## The Case of Regenerative Medicine

The field of regenerative medicine can be traced back over 30 years with earlier work in the area of tissue engineering. Regenerative medicine involves the use of tissues, cells (embryonic stem cells, induced pluripotent stem cells, and adult stem cells) or genes to treat or manage illness and disease. Its more recent developments, which have triggered so much scientific, corporate and clinical interest, relate to cell and gene therapy, both of which involve the manipulation of live tissue either *ex vivo* or *in vivo*. The advent of the technique known as “CRISPR” (clustered regularly interspaced short palindromic repeats) in the very recent past is especially important (Papdaki, 2016). This works through either repairing, augmenting “good” genes, or dis-activating (“turning-off”) or replacing dysfunctional genes that cause disease with the aim of (re)establishing normal function. Advocates proclaim that it has the potential to produce curative treatments for a range of conditions for which there is currently unmet clinical need, including cancers, and cardiovascular, neurological, and autoimmune diseases. The field, however, is still in its infancy, and the complexity of regenerative medicine technologies presents a range of scientific, technical, regulatory and reimbursement challenges for investigators and manufacturers working within the field.

In regard to scientific and technical complexities, of central importance here is how to characterize, standardize, and develop techniques to deliver cell and gene therapies. The key problem is the procuring and manipulation of *live tissue*. This is radically different from the production of small (chemical) molecules upon which conventional drug development depends. Moreover, the use of animal models to determine safety and efficacy (common to Big Pharma) is a matter of continuing debate inasmuch as they do not translate easily to *in vivo* clinical trials in humans. In addition, the body's immune response to cells that have been manipulated can be especially powerful, potentially fatal. Standardizing and stabilizing live biological processes is then of major importance in the field but is a process that confronts many unknowns: indeed, as has been argued, it is tantamount to standardizing the unknown (see Eriksson and Webster, 2008). This in turn has generated new problems for those charged with regulating the field.

Recent years have seen many attempts—in Europe, as well as in other parts of the world—to provide regulatory clarity for the regenerative medicine research industry, and to create effective structures to foster translation to the clinic. The Advanced Therapy Medicinal Products Regulation in Europe has, with some difficulty, sought to create a harmonized framework for therapies such as gene therapy, somatic cell therapy, and tissue engineering. Problems remain though about agreement on the classification of regenerative medicine products across EU Member states and tensions between the regulatory and the legal perspectives (Mahalatchimy, 2016). Gene therapy, for example, falls under the GMO (Genetically Modified Organisms) Directive which was not designed to regulate medicinal products, so in Europe novel therapies have to be submitted to authorities responsible for both GMO and for medicinal products.

Challenges to the conventional regulatory system have been echoed elsewhere. Recent research has identified a number of specific difficulties faced by the field (Gardner et al., 2015). These relate to the perceived need for bespoke clinical trials design that can manage the complications of introducing and measuring the effect of live tissue. Scaling up and manufacturing cell lines for possible therapies poses additional problems in stabilizing and quality assuring batches of tissue that can be delivered to clinics in an optimal and time-dependable way. This has led some in the field to argue for a distributed rather than centralized production and distribution system, with rapid delivery more possible. However, this in turn generates complications for quality assurance and related regulatory oversight for a system that is geared toward more centralized forms of manufacture and use.

The UK government in response recently established three new research and clinical application centers to foster and trial the use of “Advanced Therapies” within the NHS (Gardner et al., 2018). These are novel in form and purpose as sites for clinical translation, organizationally complex (bringing academic, industry and hospitals together) arrangements. The new centers will have to encapsulate and address the challenges noted above and so make regenerative medicine workable within the clinic. They are in effect pilots of central importance to the development of the field for they will have to create novel organizational structures and practices—notably in hospitals—related to the handling and delivery of live tissue. The wider context helps explain their creation inasmuch as they are seen to be test beds through which to speeden up patient access to novel therapies. Moves in the UK toward accelerated innovation led to various policy documents addressing this in general and its role in regenerative medicine. The Accelerated Access scheme (Accelerated Access Review, 2016) has introduced a radical framework through which “transformative” products are fast-tracked through approval processes−10 year are envisaged. The scheme itself can be seen to be as transformative as the products. This will be a novel and challenging cooperation to make the products workable: different forms of evidence, different interests, different infrastructures (commercial, regulatory, and clinical) and different priorities (over budgets, commissioning, skills base-needs, and so on) will need to be aligned.

## Acceleration: Regulation- and Risk-in-the-Making

The story of regenerative medicine reflects similar trends elsewhere in fast-tracking innovation, based on the perception that regulatory regimes have slowed it down. This is despite there being a considerable body of work that demonstrates how regulation is, in the medium to long term, enabling of more stable products and so the creation of new markets (Edquist et al., 2004). The question then this raises for the future is whether the products that emerge via this more iterative, yet accelerated, process will carry with them unforeseen risks-in-the making that will require novel forms of monitoring and review, and indeed how markets for innovation cope with its complexities and uncertainties.

A number of scholars have discussed the embrace of the “acceleration” in today's research and innovation system. Felt (2017) for example, shows how this creates tensions and difficulties for academics, required to switch their focus from a logic of discovery to one of “delivery” (though impact, rapid publication of papers, and meeting different indicators and metrics of worth) in rhythms of production and deadlines set by others within and outside of the university. The race to produce has been shown to have negative consequences: as “products” of research, for example, papers driven by the need to publish may not be reproducible (Begley and Ioannidis, 2015), lacking in accuracy of methods, data, and so results. Rosa (2013) has argued that “technical acceleration” has led to the acceleration of goal-directed processes driven by economic priorities. Innovation programmes and initiatives such as the new ATTCs in the UK will experience this conflation of discovery and delivery and the need to manage different logics and their associated timeframes and rhythms. In this sense we have to see “regulation” as part of a wider regime of governance and accountability located within the science production process. Products that emerge from this reflect these differing rhythms and demands and the metrics associated with each. Crucially, then, we need organizational and governance structures that understand the impact of the temporalities of acceleration and its associated measures and how the latter will depend on digital tracking systems in complex supply chains (which raise additional questions about cyber security and data safety). This also would be to recognize the *non-linear* rhythms of innovation, especially reflecting its place within socio-technical networks.

Future oversight of biomedical innovation needs to characterize the temporalities of different forms of product and therapy and the types of data they generate, and how these might be aligned. Does the existing governance regime work to identify key risks that may emerge, and who bears them (especially in the context of “personalized medicine”)? For example, as noted above, distributed vs. centralized production systems are built on radically different timeframes and supply/user chains; or, again, early access to medicines vs. at the end of a full trial (as in the Japanese case) depend on different temporal monitoring of outcomes and projections into the future based on differing evidence bases.

This isn't then a call for “slowing down”—something many have championed (Stengers, 2018)—but understanding the “timescape” (Adam, 1998) of innovation in a context where the language of acceleration and faster delivery dominates.

## Concluding points

Novel fields of inquiry and practice, such as in the field of regenerative medicine, and the complexities and attendant uncertainties and concerns that surround them, have often been the focus of debate among sociologists of science (e.g., Calvert, 2013) as well as those directly involved in trying to both promote yet oversee safe innovation (HoC, 2017). Within this wider context, we have seen the development over the past 5 years of a new paradigm through which to understand and both enable yet constrain emergent science and technologies that are shot through with uncertainty and novel risk: this is the move toward the paradigm of “responsible research and innovation.” For purposes of this Perspective commentary, we can suggest there is a need to accelerate responsibly. This would avoid the danger of moving rapidly to solutions without carefully knowing what the problem is and what success looks like, and what we need to do to adapt if things go wrong.

Using this approach, complexity as such is not completely removed: instead, some agreement can be made about how it can be handled, and to what degree some simplification of it as doable can be agreed (Mol and Law, 2002). This requires a need to retain openness, postpone lock-in, and to recognize uncertainty. Most importantly from a political perspective, it also requires a move away from a focus on the *rate* of innovation to one that attends to the *direction* of innovation itself.

## Author Contributions

The author confirms being the sole contributor of this work and has approved it for publication.

### Conflict of Interest Statement

The author declares that the research was conducted in the absence of any commercial or financial relationships that could be construed as a potential conflict of interest. The handling editor declared a shared affiliation, though no other collaboration, with the author at the time of review.
